# Antibiotic Prescribing and Use in Hospitals and Long-term Care

**DOI:** 10.1093/jacamr/dlz004

**Published:** 2019-04-08

## Abstract

**Graphical Abstract:**
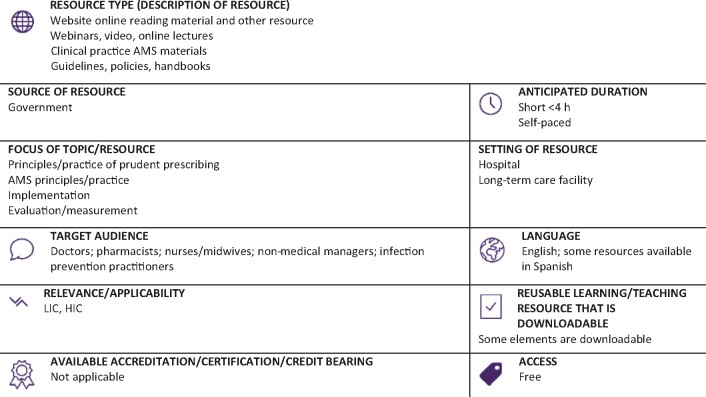

**Resource web link: https://www.cdc.gov/antibiotic-use/healthcare/programs.html** (Full classification scheme available at: http://bsac.org.uk/wp-content/uploads/2019/03/Educational-resource-review-classification-scheme.pdf)

**WHO region and country (World Bank):** North America, USA (HIC)

## Peer review commentary

This is a website which is mostly hosting links to a number of websites of North American healthcare institutions that have antimicrobial stewardship (AMS) programmes; these are mostly organized in the same way, with a strong emphasis on the traditional prescriber-pharmacist interactions for improving prescribing. There are case studies of successful AMS programmes in a variety of institutions, which might provide inspiration for those looking to implement an AMS programme in a healthcare setting which models these. There are useful summary tables of evidence/publications that demonstrate the impact of AMS interventions in reducing resistance, costs etc., but these are in need of updating with more recent references. Potentially the most useful part of this website for users from less resourced countries is a PDF document discussing the core elements of AMS programmes in resource-limited settings.

Overall, this is a resource that will suit users who enjoy reading information and who would be inspired by examples from other healthcare institutions; there is no interactive content and limited educational content. However, if users are looking for primers on organizing and setting up formal AMS programmes, this is a good place to start.

